# Complete Genome Sequence of Serratia marcescens Siphophage Scapp

**DOI:** 10.1128/MRA.00417-19

**Published:** 2019-05-09

**Authors:** Brian T. Koehler, Helena Hopson, Rohit Kongari, Rachele Bonasera, Adriana C. Hernandez-Morales, Mei Liu

**Affiliations:** a Center for Phage Technology, Texas A&M University, College Station, Texas, USA; Queens College

## Abstract

Serratia marcescens is an opportunistic pathogen that typically infects the respiratory and urinary tract, with the majority of cases being hospital acquired. The study of S. marcescens phages may help control drug-resistant S. marcescens strains. In this study, we announce the complete genome sequence and the features of S. marcescens siphophage Scapp.

## Abstract (precis)

Serratia marcescens is an opportunistic pathogen that typically infects the respiratory and urinary tract, with the majority of cases being hospital acquired. The study of S. marcescens phages may help control drug-resistant S. marcescens strains.

## ANNOUNCEMENT

Serratia marcescens is an opportunistic pathogen that typically infects the respiratory and urinary tract, with the majority of cases being hospital acquired (1–3). Many strains of S. marcescens identified in intensive care unit patients in U.S. hospitals possess resistance to most available antibiotics (4). The study of S. marcescens phages may help control drug-resistant S. marcescens strains.

The siphophage Scapp was isolated using an S. marcescens strain from activated sludge collected from the water treatment plant in College Station, TX. Host bacteria were cultured on nutrient broth or agar (Difco) at 37°C with aeration. Phages were isolated and propagated by the soft agar overlay method (5). Phage genomic DNA was prepared using a modified Promega Wizard DNA cleanup kit protocol, as described previously (6). Pooled indexed DNA libraries were prepared using the Illumina TruSeq Nano low-throughput (LT) kit, and the sequence was obtained by the Illumina MiSeq platform using the MiSeq v2 500-cycle reagent kit, following the manufacturer’s instructions, producing 447,621 paired-end reads for the index containing the phage genome. The quality of the reads was checked in FastQC 0.11.5 (https://www.bioinformatics.babraham.ac.uk/projects/fastqc/), and reads were trimmed with the FastX-Toolkit 0.0.14 (http://hannonlab.cshl.edu/fastx_toolkit/) and assembled in SPAdes 3.5.0 (7). The assembled genome was closed by PCR using primers 5′-AAACAACGGAGTGGGAAGAG-3′ and 5’-CAGGGTCTATCACGCAGTAAAT-3′ facing away from the center of the assembled contig and by Sanger sequencing of the resulting product, with the contig sequence manually corrected to match the resulting Sanger sequencing read. Protein-coding genes were predicted using Glimmer 3.0 (8) and MetaGeneAnnotator 1.0 (9) and corrected manually if needed. The tRNA genes were predicted using ARAGORN 2.36 (10). Protein functions were predicted by comparing sequence homology to proteins found using BLASTp 2.2.28 (11), and conserved domains were analyzed using InterProScan 5.15-5.40 (12). All analyses were performed under default settings using the CPT Galaxy (13) and WebApollo (14) interfaces (cpt.tamu.edu).

The complete 42,969-bp Scapp genome was assembled at 192.1-fold coverage. It has 59 protein-coding genes, an overall coding density of 93%, and a GC content of 56%. Using the progressiveMAUVE algorithm (v2.4.0) (15), Scapp shows less than 20% DNA sequence similarity to any other phage in the NCBI nucleotide (nt) database. The Scapp genome begins with an ∼6,000-bp region that contains novel genes with no sequence similarity to other proteins in the NCBI nonredundant (nr) database. At the protein level, phage Scapp is most closely related to phages APSE-2 (GenBank accession number EU794049) and a prophage-like element (GenBank accession number HQ377374) associated with insect symbionts. Some of the structural proteins of phage Scapp are related to common enterobacterial siphophage proteins (BLASTp E value, ≤10^−3^), such as those found in phages N15, T1, and Lambda. These genes include those encoding head assembly, major capsid, tape measure, major tail, tail tip, and four minor tail proteins. An endonuclease, a transcriptional regulator, and a transposase are located next to the lysis cassette but do not interrupt any genes. An adjacent holin-antiholin pair and an endolysin (d-alanyl-d-alanine carboxypeptidase) were identified. The o-spanin is embedded in the i-spanin, and this spanin complex is located separately from the other lysis genes.

### Data availability.

The genome sequence of phage Scapp was deposited under GenBank accession number MH553517. The associated BioProject, SRA, and BioSample accession numbers are PRJNA222858, SRR8788475, and SAMN11259833, respectively.
